# A dose-reduction HPV vaccine immunobridging trial of two HPV vaccines among adolescent girls in Tanzania (the DoRIS trial) – Study protocol for a randomised controlled trial

**DOI:** 10.1016/j.cct.2021.106266

**Published:** 2021-02

**Authors:** Kathy J. Baisley, Hilary S. Whitworth, John Changalucha, Ligia Pinto, Joakim Dillner, Saidi Kapiga, Silvia de Sanjosé, Philippe Mayaud, Richard J. Hayes, Charles J. Lacey, Deborah Watson-Jones

**Affiliations:** a MRC Tropical Epidemiology Group, Department of Infectious Disease Epidemiology, London School of Hygiene and Tropical Medicine, Keppel Street, London, UK; b Africa Health Research Institute, KwaZulu-Natal, South Africa; c Clinical Research Department, London School of Hygiene and Tropical Medicine, Keppel Street, London, UK; d Mwanza Intervention Trials Unit, National Institute for Medical Research, Mwanza, Tanzania; e Vaccine, Immunity and Cancer Program, HPV Immunology Laboratory, Leidos Biomedical Research Inc, Frederick National Laboratory, Frederick, MD, USA; f Department of Laboratory Medicine, Karolinska Institutet, Stockholm, Sweden; g PATH, Seattle, WA, USA; h National Cancer Institute, Rockville, MD, USA; i York Biomedical Research Institute, Hull York Medical School, University of York, UK

**Keywords:** Probiotic, Olive flounder, Microbiota, Growth performance

## Abstract

**Background:**

Human papillomavirus (HPV) infection is the primary cause of cervical cancer. In 2018, the World Health Organization (WHO) Director General announced his commitment to eliminate cervical cancer, with HPV vaccination as a priority. However, the costs of setting up a multi-dose HPV vaccination programme remain a barrier to its introduction.

**Methods/Design:**

We are conducting a randomised-controlled trial of reduced dose schedules of HPV vaccine in Tanzania to establish whether a single dose produces immune responses that will be effective in preventing cervical cancer. 930 girls aged 9–14 years in Mwanza, Tanzania, were randomised to one of 6 arms, comprising 3 different dose schedules of the 2-valent (Cervarix) and 9-valent (Gardasil-9) HPV vaccines: 3 doses; 2 doses given 6 months apart; or a single dose. All participants will be followed for 36 months; those in the 1 and 2 dose arms will be followed for 60 months. Trial outcomes focus on vaccine immune responses including HPV 16/18-specific antibody levels, antibody avidity, and memory B cell responses. Results will be immunobridged to historical cohorts of girls and young women in whom efficacy has been demonstrated.

**Discussion:**

This is the first randomised trial of the single dose HPV vaccine schedule in the target age group. The trial will allow us to examine the quality and durability of immune responses of reduced dose schedules in a population with high burden of malaria and other infections that may affect vaccine immune responses. Initial results (24 months) are expected to be published in early 2021.

## 1 Background

Human papillomavirus (HPV) infection, the primary cause of cervical cancer, is a major public health problem in sub-Saharan Africa (SSA). East Africa has an estimated cervical cancer incidence of around 40/100,000 [1], among the highest in the world. In many countries in SSA, screening is absent or limited, and treatment is often sub-optimal.

In 2018, the Director-General of the World Health Organization (WHO) announced his commitment to eliminate cervical cancer [2]. Prophylactic HPV vaccines, critical for this elimination goal, are safe and highly effective at preventing HPV infection and associated disease. Three HPV vaccines are licensed; the bivalent vaccine protects against HPV 16/18 (Cervarix®), the 4-valent vaccine against HPV 6/11/16/18 (Gardasil®), and the 9-valent vaccine against 9 genotypes (HPV 6/11/16/18/31/33/45/52/58) (Gardasil-9®).

The vaccine was originally given as a 3-dose schedule. However, a 2-dose schedule was approved in 2016 for girls aged <15 years [3]. Of 127 countries that had included HPV vaccines in their national programmes by May 2020, only 22 are low- or middle-income countries (LMIC) [4]. The costs of setting up and sustaining a multi-dose HPV vaccine programme remain a barrier to its introduction [5,6]. Cost and logistics have also limited the implementation of extended age range ‘catch-up’ campaigns in existing programmes [7]. New vaccination strategies are therefore needed to enable cervical cancer elimination. A 1-dose schedule could reduce costs and simplify vaccine delivery, facilitate rollout of national programmes and catch-up campaigns, and dramatically reduce the cervical cancer burden globally.

Data suggest that 1 dose of HPV vaccine may confer sufficient protection against HPV infection and cervical cancer precursors. Women who received 1 or 2 doses of Cervarix® in the Costa Rica Vaccine (CVT) and PATRICIA trials (due to non-completion of the 3-dose schedule) had similar efficacy against HPV infection over 4 years of follow-up compared with those who received 3 doses [8]. Women who received fewer than 3 doses in the CVT are being followed long-term, and the 11-year efficacy and immunogenicity data support durable protection from 1 dose [9]. Furthermore, 1 dose provided antibody levels well above those found following natural infection. A trial of Gardasil® in India found that participants who received only 1 dose had similar incident and persistent HPV infections over 7 years as those receiving 3 doses [10]. Whilst these results challenge the established belief that protein-based subunit vaccines require a prime-boost regimen, they provide insufficient evidence to change vaccine recommendations because of their non-randomised design and post-hoc character.

The 2-dose schedule in girls aged <15 years was approved based on immunogenicity studies in high and upper middle-income countries. However, it is conceivable that the efficacy of reduced-dose schedules could be affected by intercurrent infections such as helminths or malaria [11]. We are conducting a randomised-controlled trial of reduced dose schedules of 2 HPV vaccines in Tanzania, to establish whether 1 dose produces immune responses that are likely to be effective in preventing cervical cancer in SSA. This is the first randomised trial of the single dose schedule in 9 to 14 year-old girls, the primary target group for this vaccine globally.

### 1.1 Trial objectives and outcomes

The overall objective of this trial is to determine whether a single dose of the bivalent vaccine (Cervarix®) or 9-valent vaccine (Gardasil-9®) produces immune responses that are non-inferior to those following 2 and 3 doses when given to HIV negative girls aged 9 to 14 years in a malaria-endemic region of Tanzania, and whether these immune responses are affected by malaria infection. We will also compare immune responses after 1 dose in young girls in Tanzania with those in historical cohorts of girls and young women who received 1, 2 or 3 doses of HPV vaccine, in whom efficacy has been demonstrated.

The trial outcomes focus on vaccine immune responses as measured by: (1) the proportion of participants seroconverting to HPV types 16/18; (2) geometric mean titre (GMT) of HPV 16/18-specific antibodies; (3) HPV 16/18-specific antibody avidity; and (4) HPV 16/18-specific memory B cell responses.

The trial has two co-primary objectives: 1) to demonstrate non-inferiority of HPV 16/18-specific seropositivity following 1 dose of HPV vaccine compared with 2 or 3 doses of the same vaccine at month (M)24; and 2) to demonstrate non-inferiority of antibody GMT at M24, when comparing the 1 dose regimen of either vaccine with historical cohorts of women aged 10–25 years who received 1 dose, in whom efficacy has been demonstrated. Secondary immunogenicity objectives include evaluation of HPV 16/18 antibody GMT and seropositivity at other timepoints, evaluation of antibody avidity and memory B cell responses, comparison of immune responses after 2 versus 3 doses, comparisons of the same dose regimen between vaccine types, and comparisons between girls who had malaria at the time of vaccination and those who did not. The primary focus is on HPV16/18; however, the antibody response to the other HPV genotypes covered by the 9-valent vaccine will also be explored. Other secondary objectives are evaluating cost effectiveness and acceptability of the 1 dose schedule.

Girls in the 1- and 2-dose arms will be invited to enrol in a trial extension, to examine the durability and stability of immune responses up to 60 months. The primary objective of the trial extension is to demonstrate non-inferiority of HPV 16/18-specific seropositivity when comparing 1 dose with 2 doses of the same vaccine at M60.

## 2 Methods

### 2.1 Study design and population

This is an open-label, individually-randomised controlled trial of two HPV vaccines being conducted at the Mwanza Intervention Trials Unit (MITU), in the lake zone region of north-western Tanzania [Dose Reduction Immunobridging and Safety Study of two HPV vaccines in Tanzanian girls (DoRIS); NCT02834637].

The trial has 6 arms comprising 3 different dose schedules of the bivalent or 9-valent HPV vaccines: the originally recommended 3 dose schedule; 2 doses given 6 months apart; or a single dose (Table 1). All girls will be followed for 36 months; those who consent to the extension will be followed for 60 months.

**Table 1:** Design of the trial Dose Reduction Immunobridging and Safety Study of two HPV vaccines in Tanzanian girls (DoRIS).

Arm	2-valent HPV vaccine (Cervarix®)			9-valent HPV vaccine (Gardasil-9®)			Total
	3 doses^a^	2 doses^b^	1 dose	3 doses^c^	2 doses^b^	1 dose	
	A	B	C	D	E	F	
Number of girls	155	155	155	155	155	155	930

a Given at Day(D)0, Month(M) 1 and M6.

b Given at D0 and M6.

c Given at D0, M2 and M6.

The trial has enrolled 930 HIV-negative schoolgirls living in Mwanza. Enrolment began in March 2017 and ended in January 2018; follow-up is expected to end in May/June 2021 for the main trial (owing to SARS-CoV-2 outbreak and postponement of some M36 visits) or January/February 2023 for the extension. Girls were eligible for inclusion in the main trial if aged 9–14 years, planning to be resident in Mwanza for 36 months and willing and able to give informed assent, with informed consent from parent/guardian. Girls were excluded if they had previously received any dose of HPV vaccination, had a past history of cervical lesions or genital warts, had received treatment for positive cervical screening, were pregnant at screening, were immunocompromised, including HIV infection, or were unwell on the basis of medical history, clinical examination or laboratory tests. At the M36 visit, all girls in the 1 and 2 dose arms will be invited to participate in the trial extension.

### 2.2 Randomisation

Eligible participants were randomised to one of the 6 study arms in a 1:1:1:1:1:1 allocation, using random permuted block sizes of 12, 18 and 24. The randomisation list was computer-generated by an independent statistician, with the treatment allocation order defined by the blocks and sequence within blocks. Trial participant identification numbers were generated within the computer program, and sequentially assigned in the order of the treatment allocations.

A set of sequentially-numbered opaque sealed envelopes, each containing a unique participant identification number with its allocation, were prepared by the independent statistician in advance of the enrolment visit and sent to the research clinic. At the enrolment visit, after eligibility was confirmed, the study clinician responsible for enrolment opened the next available envelope in the numbered sequence in order to find the participant's identification number and assigned allocation. The identification number and allocation were recorded on the participant's case report form (CRF).

### 2.3 Sample size

With the 2- and 3-dose schedules of either HPV vaccine, it is estimated that 99% will be seropositive for HPV16/18 at M24 [12]. With 155 in each HPV-dose schedule arm, assuming <5% have HPV 16/18 antibodies or are HPV 16/18 DNA positive at enrolment (based on our previous studies in Tanzania), [[13], [14], [15]] and a projected 20% loss to follow up (LTFU) over 36 months, we expect to have around 130 girls in each arm at the M24 visit for the primary outcome analyses, 120 girls at M36, and 100 at M60.

If the true proportion seroconverting is the same in each arm, with 130 girls per arm, we will have >90% power to conclude that seropositivity with the reduced dose schedule is not decreased by more than 5.0%, using a one-sided non-inferiority test at the 2.5% level (Table 2). This non-inferiority margin is the same that was used in the trials leading to licensure of the 2-dose regimen in girls aged <15 years [16]. If the true GMT ratio (reduced dose arm: comparison cohort) between groups is 1.0, with 130 girls in each group, we will have >90% power to conclude that the reduced dose schedule does not decrease anti-HPV 16/18 GMT by more than 50%, corresponding with a reduction of 0.30 in log titre. The non-inferiority margin was based on pre-established standards from the US Food and Drug Administration (FDA) that have been used in other HPV vaccine bridging trials [16,17]. We have assumed an SD of 0.50–0.60 log10 anti-HPV titre [12], and used a one-sided non-inferiority test at the 2.5% level.

**Table 2:** Non-inferiority margins that can be demonstrated with 90% power, for different assumptions of number of subjects evaluable in each arm.

Outcome	Number evaluable^a^	True value in population	Non-inferiority margin^b^	Power
HPV 16/18 proportion seroconverting	130	99%	4.0%	90%
	100	99%	4.6%	90%
	85	99%	5.0%	90%
HPV 16/18 antibody GMT ratio^c^	130	1.0	0.57	90%
	100	1.0	0.53	90%
	85	1.0	0.50	90%

a Evaluable subjects are those attending at M24, M36 or M60 who are HPV 16/18 DNA and antibody negative at enrolment.

b Non-inferiority defined as lower bound for 95% 2-sided CI for difference in proportions/ratio of GMT being above this margin.

c Assuming an SD of 0.60 log10 anti-HPV titre.

### 2.4 Study interventions

Both vaccines used in this trial are licensed by the US Food and Drug Administration (FDA). The bivalent HPV vaccine (Cervarix®), produced by GSK Biologicals, contains HPV 16/18 virus-like-particles (VLP). The 9 valent vaccine is produced by Merck (Gardasil-9®) and contains HPV 6, 11, 16, 18, 31, 33, 45, 52 and 58 VLPs. The bivalent vaccine has an adjuvant consisting of monophosphoryl lipid A (MPL) and aluminium hydroxide. MPL is a detoxified bacterial lipopolysaccharide which is a TLR-4 agonist which causes activation of both innate and adaptive immune responses [18] The 9-valent vaccine uses a more traditional aluminium adjuvant (aluminium hydroxyl-phosphate sulphate), similar to that of the 4-valent vaccine, but in a higher dose. Antibody levels produced by the bivalent vaccine are significantly higher than those produced by the 4-valent vaccine both for HPV 16 and 18 and for cross-protected types [19].

## 3 Study procedures

### 3.1 Preparatory activities

Girls were enrolled from 36 primary and 18 secondary government day schools in Ilemela municipality, Mwanza city. In the month before the screening visit, study mobilisers held meetings with community and religious leaders and heads of schools to explain the trial. Parents/guardians of potentially eligible girls attending the selected schools were invited to a meeting at the school where the trial and informed consent and assent procedures were explained. Parents/guardians were then approached individually and invited to attend the research clinic with their daughters for screening.

### 3.2 Screening and enrolment

A summary of the study procedures is shown in Table 3. At the screening visit, girls had the trial aims, eligibility criteria and procedures explained. Parents/guardians and girls were asked for their informed written/witnessed consent and assent, respectively. All girls aged ≥12 years were required to pass a Test of Understanding (TOU) in order to be eligible for enrolment; for younger girls, the parent/guardian was required to pass the TOU. Parents/guardians and girls were allowed to retake the test twice if they failed to reach the pass score of >90%. If the TOU was passed, girls were screened for eligibility, including a medical history with a physical examination if indicated, HIV counselling and testing and a urine test was performed for pregnancy. Girls who were HIV positive were not eligible for enrolment, but were encouraged to share the test result with their parent/guardian, and were referred for CD4 count assessment and HIV care. Girls who were found to be pregnant at the screening or enrolment visit were considered to be a screening failure and were also ineligible.

**Table 3:** Summary of study procedures.

Study procedure	Screen < 30 d	D0	M1	M2	M3	M6	M7	M12	M18	M24	M30	M36	M42^g^	M48^g^	M54^g^	M60^g^
Informed consent/assent	X															
Informed consent/assent for trial extension												X^g^				
Demographics & tracing info	X											X^g^				
Medical history^a^	X															
Test of Understanding	X															
Blood sampling for HIV	X															
Pregnancy test	X	X	X^b^	X^b^		X^b^										
Check LMP & pregnancy test if indicated			X^c^	X^c^	X	X^c^	X	X		X		X		X		X
Eligibility check	X	X														
Clinic visit	X	X	X	X^d^	X^d^	X	X	X		X		X		X		X
Home, clinic or school visit									X		X		X		X	
Blood sampling for immunogenicity		X	X				X	X		X		X				X
Vaginal swabs for HPV genotyping		X														
Blood sampling for malaria		X	X^b^	X^b^		X										
Blood sampling for HSV-2		X					X^e^	X^e^		X^e^		X^e^				X
Review of medical history^a^		X	X^b^	X^b^		X^b^										
Check deferral criteria and contraindications		X	X^b^	X^b^		X^b^										
Vaccine administration		X	X^b^	X^b^		X^b^										
Recording of AEs in 30 days post vaccination			X	X^f^	X^f^		X^f^									
Recording of unsolicited AEs/SAE		X	X	X	X	X	X	X	X	X	X	X	X	X	X	X

Timing of laboratory assays																
HPV antibody ELISA		X	X				X	X		X		X				X
HPV antibody avidity								X		X		X				
Memory B cells		X	X				X	X		X		X				
PSV Luminex assay		X	X				X	X		X		X				X
Malaria		X	X^b^	X^b^		X										
HSV-2		X					X^e^	X^e^		X^e^		X^e^				X
HIV	X															

a Examination if warranted.

b Only for those randomised to vaccine at that visit.

c For those not randomised to receive vaccine at that visit.

d M2 visit attended by 3 dose arms only; M3 visit attended by 3 dose Gardasil-9 arm only.

e Storage of serum sample for HSV-2 serology at last visit attended.

f Questions about AEs that occurred in the 30 days since the last dose, only for those participants who received vaccine at the previous visit.

g Extension activities will be conducted for girls in 1 and 2 dose arms only.

The enrolment visit was within 30 days after screening. A brief interview was conducted, another urine pregnancy test was done, and eligibility criteria were re-confirmed by the study clinician. If deemed eligible, the participant was enrolled and randomised to receive the first dose of vaccine. Girls who were ineligible because of medical history and/or physical examination were referred to a doctor for appropriate medical management according to local treatment guidelines.

Digital fingerprints were taken in order to confirm a participant's identity throughout the study. The fingerprint record was stored electronically and linked only to the participant identification number, not to the participant's name or any personal identifiers. Each participant was also given a study photo identification (ID) card. Before the first dose was given, a venous blood sample was collected for immunogenicity assays, HSV-2 serology, and a dried blood spot (DBS) for storage for malaria testing by polymerase chain reaction (PCR). Two nurse-assisted, self-administered vaginal swabs were collected for HPV DNA testing and HPV genotyping. This method of sample collection has been successfully used in other studies of HPV in girls in Mwanza [14,15].

### 3.3 Vaccination phase

Vaccination was conducted at enrolment (Day(D) 0; all arms), M1 or M2 (3-dose arms; Cervarix and Gardasil-9, respectively) and M6 (2- and 3-dose arms). A short medical history and repeat urine pregnancy test was done at each vaccination visit prior to vaccination. Vaccination was postponed if the girl was deemed to have an acute illness that precluded vaccination. Girls who were pregnant did not receive any further doses of vaccine but continued with the study follow-up visits.

Vaccines were administered via intramuscular injection into the deltoid region of the upper arm. After vaccination, participants were observed for at least 30 min, with appropriate medical treatment and equipment available in case of any anaphylactic reaction. The study staff monitored the participant's vital signs and looked for injection site reactions and other adverse events, which were recorded on the CRF. At each vaccination visit, a blood sample was collected for a DBS, which was stored for malaria PCR testing.

Participants attended the clinic one month after each vaccination visit for questioning about adverse events in the 30 days after vaccination, and for blood sampling for immunogenicity outcomes. The windows for vaccination and blood sampling are in Table 4.

**Table 4:** Window periods for vaccination and follow-up visits.

Procedure	Arms^a^	Visit	Recommended date	Minimum date	Maximum date
Vaccination visits					
Dose 1	All	D0	Date of first vaccination (DFV)	N/A	N/A
Dose 2	A	M1	DFV +30 days	DFV +30 days	DFV +60 days
Dose 2	D	M2	DFV +60 days	DFV +30 days	DFV +90 days
Dose 2	B and E	M6	DFV +181 days	DFV +181 days	DFV +271 days^b^
Dose 3	A and D	M6	DFV +181 days	DFV +181 days	DFV +271 days^b^

Follow-up visits					
D0 blood sample	All	D0	DFV	N/A	N/A
M1 blood sample and AE recording^c^	All	M1	DFV +30 days	DFV +30 days	DFV +60 days
M2 AE recording^c^	A	M2	Date of second vaccination +30 days	Date of second vaccination +30 days	Date of second vaccination +60 days
M2 blood sample for malaria	D	M2	Date of second vaccination	N/A	N/A
M3 AE recording^c^	D	M3	Date of second vaccination +30 days	Date of second vaccination +30 days	Date of second vaccination +60 days
M6 blood sample for malaria	A, B, D and E	M6	Date of last vaccination	Date of last vaccination	Date of last vaccination
M6 blood sample for malaria	C and F	M6	DFV +181 days	DFV +181 days	DFV +211 days
M7 blood sample	A, B, D and E	M7	Date of last vaccination +30 days	Date of last vaccination +30 days	Date of last vaccination +60 days
M7 blood sample	C and F	M7	DFV +211 days	DFV +211 days	DFV +241 days
M12 blood sample	All	M12	DFV +361 days	DFV +361 days	DFV +391 days
M24 blood sample	All	M24	DFV +723 days	DFV +723 days	DFV +753 days
M36 blood sample	All	M36	DFV +1085 days	DFV +1055 days	DFV +1265 days^d^
M60 blood sample	B, C, E, F	M60	DFV +1809 days	DFV +1779 days	DFV +1839 days

a Arms A, B and C receive 3 doses, 2 doses and 1 doses of Cervarix®, respectively. Arms D, E and F receive 3 doses, 2 doses and 1 doses of Gardasil-9®, respectively.

b For ethical reasons, girls may receive the last dose up to 360 days after DFV; however, they may be excluded from the immunogenicity analysis.

c Solicited signs and symptoms in the 30 days after each vaccine dose.

d Window extended because of SARS-CoV-2 outbreak and postponement of some M36 visits.

### 3.4 Follow-up

All participants were asked to attend the clinic at M6 and M7 to collect a blood sample for a DBS for malaria PCR (M6) and for vaccine immunogenicity assays (M7). Scheduled follow-up visits are at M12, M24 and M36, and a blood sample is collected for immunogenicity. Participants in the trial extension will also be followed up at M48 and M60, and an immunogenicity blood sample will be collected at M60.

At M18 and M30 (and M42 and M54 in the trial extension), participants are visited at home or at school to ensure that they are still living in Mwanza and to update contact details if needed. Participants are questioned about AEs at all study visits. In addition, to help ensure a high rate of retention between visits, participants are sent an SMS reminder or telephoned about the trial every 3 months.

In April 2020, the trial was temporarily suspended owing to the SARS-CoV-2 outbreak; a protocol amendment was submitted to increase the window for the M36 blood sample (Table 4). The Tanzanian National Health Research Ethics Sub-Committee (*NatHREC*) gave permission for studies to resume activities as per protocol on 18 May 2020 provided that training is done in small groups and COVID-19 preventative measures, such as mask-wearing, physical distancing and hand hygiene, are implemented for research activities. The trial team resumed month 36 visits on 3 August 2020.

### 3.5 Laboratory assays

Whole blood samples of up to 20 mL (depending on girl's weight) are collected for immunological assays, in order to provide 10 mL of serum and 10 mL for peripheral blood mononuclear cells (PBMC). All samples are processed and stored initially at the laboratory at the National Institute of Medical Research (NIMR) in Mwanza, before being shipped to the relevant laboratory for analysis. HPV 16/18 antibodies will be measured by a qualified anti-VLP ELISA assay at the Frederick National Laboratory for Cancer Research HPV Immunology Laboratory in Maryland, USA [20]. The primary analyses will be based on antibody GMT as measured in this VLP ELISA. The HPV 16/18-specific antibody avidity index (AI) will be determined in the ELISA by the ratio of antibody concentrations in serum samples treated or not treated with Guanidine-HCl [21]. HPV 16/18-specific memory B cell responses will be measured in PBMCs by a B cell ELISPOT assay at the Centre for Immunology and Infection, York, UK; detectable HPV type-specific memory B-cells will be defined as >1 antigen-specific memory B cell/million memory B cells [22]. Serum HPV antibody titres to the HPV genotypes in the 9-valent vaccine are being measured by a pseudovirion (PsV)-based antibody Luminex assay at the Karolinska Institute, Sweden; the assay has shown high correlations with VLP-ELISA and neutralisation assays, and with natural infection [[23], [24], [25]]. HPV DNA genotyping at D0 was done using the Anyplex HPV28 (Seegene, South Korea) detection assay at the Catalan Institute of Oncology, Barcelona.

### 3.6 Data management

All completed CRFs and laboratory forms are submitted to the MITU data section. Data are double-entered into a study-specific database by trained data entry staff, using the OpenClinica open source software. Data checks and data cleaning are done by trained data managers at MITU under the supervision of a senior data manager. Submitted CRFs and forms are stored securely in locked filing cabinets in the MITU data department. At the conclusion of the study, the database will be archived in accordance with internal procedures.

### 3.7 Statistical analysis

In non-inferiority trials, intention-to-treat (ITT) analyses may increase the risk of falsely claiming non-inferiority, since these analyses often lead to smaller observed effects than if all participants had adhered to the protocol [26]. Therefore, for the non-inferiority objectives, we will conduct the primary analyses in the per-protocol (PP) population, and repeat all analyses in the intention-to-treat (ITT) population as a sensitivity analysis. The PP population will be girls who receive the allocated doses of vaccine within the specified windows in Table 4 and are HPV antibody and DNA negative at enrolment for the specific genotype under analysis.

Seropositivity for a particular HPV vaccine genotype will be defined as antibody level above the assay cut-off; the cut-off value will be defined by the laboratory before the analysis begins. We will measure the proportion of girls in each arm who are seropositive for each HPV vaccine genotype, and calculate the difference (reduced dose group minus comparison group) between arms. We will estimate the 95% CI for the difference using the Farrington and Manning approach [27]. Non-inferiority will be concluded if the lower 95% CI for the difference is above −5%.

The ratio of HPV genotype-specific GMTs will be obtained from an analysis of variance (ANOVA) of log10 antibody titres as the response variable. Separate analyses will be done for each vaccine genotype and time point. The ANOVA model will include trial group as a fixed effect. For each vaccine type (Cervarix and Gardasil-9), a contrast will be derived from the ANOVA model comparing the mean of log10 titre in the reduced dose group with that in the comparison group, using the residual error from the ANOVA. The GMT ratio and its 95% CI will be derived from back-transformation of the mean and 95% CI from this contrast. Non-inferiority will be concluded if the lower bound for the 95% two-sided CI for the GMT ratio (reduced dose group divided by comparison group) is above 0.50.

The primary analyses will exclude girls with missing immunogenicity results (complete case), but a sensitivity analysis using multiple imputation of missing data may be done at month 36, and/or month 60.

For the primary objectives, non-inferiority for each vaccine type will be concluded if the lower limit for the 95% CI for the seroconversion difference between 1 dose vs 2 doses, and between 1 dose vs 3 doses, is above −5% for both HPV16 and HPV18. For comparisons with historical cohorts, non-inferiority for each vaccine type will be concluded if the lower limit of the 95% CI is for the GMT ratio (1D/historical cohort) is above 0.50 for both HPV16 and HPV18. For the secondary objectives, a hierarchy of testing for the non-inferiority objectives will be pre-specified in a statistical analysis plan.

A subgroup analysis will be done to compare immune responses between girls who were positive and negative for malaria at vaccination. Since malaria is measured at different timepoints relative to enrolment depending on arm, these will primarily focus on comparisons within arm, or between the same dose regimens (e.g. 1 dose of bivalent vs. 1 dose of 9-valent).

Full details of the statistical methods will be covered in a formal Statistical Analysis Plan that will incorporate a formal plan for the immunobridging analyses. For the secondary objectives, a hierarchy of testing for the non-inferiority objectives will be pre-specified. The analysis plan will also include pre-specified criteria for non-inferiority, plans for adjustment for multiplicity, and other statistical considerations for non-inferiority trials with immunogenicity endpoints, as outlined by Liu et al. [28] The final analysis plan will be approved by the Independent Data Safety and Monitoring Board (IDSMB), the Trial Steering Committee (TSC), and the Principal Investigators before the randomisation code is released and the data are analysed.

### 3.8 Immunobridging

We will bridge our results to historical cohorts of girls and young women aged 10–25 years who received 1, 2 or 3 doses, in whom efficacy has been demonstrated. These include the previous trial of the bivalent vaccine in Costa Rica, the CVT (NCT00128661), which vaccinated young women aged 18–25 years [29], and the International Agency for Research on Cancer (IARC) trial of the 4-valent vaccine in India (NCT00923702), which vaccinated girls and young women aged 10–18 years [10]. In addition, we will bridge our immunogenicity results to those of the National Cancer Institute's (NCI) large randomised controlled trial to evaluate the efficacy of the bivalent and 9-valent vaccines given as 1 or 2 doses to girls aged 12–16 years in Costa Rica (the ESCUDDO trial; NCT03180034) and with which our trial protocol has been harmonised. Bridging with a recently started efficacy trial in Kenya in young women aged 15–20 (KEN-SHE; NCT03675256) is also planned. Results from these trials are expected in 2023 (KEN-SHE) and 2025 (ESCUDDO).

### 3.9 Ethics and oversight

The trial protocol was approved by the ethical committee of the Medical Research Coordinating Committee, Tanzania (NIMR/HQ/R.8A/Vol.IX/2236), and the London School of Hygiene and Tropical Medicine (11568). Written informed consent is obtained from parents/guardians, with written assent from participants. A TSC and IDSMB were established to monitor trial progress. A community advisory board (CAB) comprising parents, teachers and other community members was established to advise the research team. Trial monitoring is being done by independent trial monitors from Kenya Medical Research Institute (KEMRI) in Kenya.

## 4 Discussion

This is the first randomised trial of the single dose schedule of HPV vaccine in 9–14 year old girls, the primary target group for HPV vaccine globally, and the first randomised trial of HPV vaccine dose reduction in SSA. Final results from the main trial are expected in early 2022, with interim results submitted for publication in early 2021. Other trials evaluating single dose protection have recently begun in Costa Rica, The Gambia and Kenya, with results available at the end of 2022 or later. These trials are all complementary, examining single-dose HPV vaccination for girls, adolescents, and young women aged 4–20 years, and address different scientific and programmatic questions.

In addition to comparisons between trial arms, our trial will compare vaccine-induced HPV-specific immune responses in young girls in Tanzania with those in historical cohorts of girls and young women who received 1, 2 or 3 doses of HPV vaccine, in whom efficacy has been demonstrated. We will also bridge our results with those from ongoing efficacy trials in Costa Rica and Kenya; our trial protocol is also harmonised with that of the ongoing trial in Costa Rica to maximise comparability between the 2 trials. Since it is difficult to evaluate HPV vaccine efficacy in young girls because of the time needed to accrue endpoints, immunobridging studies are used to infer protection when efficacy has been demonstrated in another population [30].

The true immunological correlates of protection for HPV vaccines have not yet been established. Age is a key determinant of antibody responses following HPV vaccination, with young girls having significantly higher antibody GMTs than young women [12,17]. Although the 2 dose regimen in girls aged <15 years has been approved based on vaccine-specific antibody levels, there is increasing recognition that vaccine efficacy depends on both quantity and quality of antibodies induced by the vaccine. Quality, measured by avidity of antibodies for the antigen, depends on priming of B cells which produce antibodies with different affinities for antigen. It is not known whether antibody affinity, memory B cell responses and durability of protection with fewer doses of HPV vaccine may be affected by intercurrent infections such as malaria or helminths. Our previous trial of 3 doses of the bivalent HPV vaccine in Tanzania found that girls who had malaria at the time of vaccination had significantly higher HPV 16/18 antibody levels one month after the last dose compared with girls who did not have malaria [31]. Malaria induces polyclonal antibodies which may enhance vaccine-induced anti-VLP antibodies, but the quality of these antibodies related to vaccine-induced protection is not known [32]. Ours, and the other ongoing one-dose trials, will help provide definitive answers to questions about non-inferiority of 1 dose of HPV vaccine compared with 2 doses, in terms of immunogenicity and HPV infection, and the feasibility of dose reduction.

Following the call from the WHO Director General in 2018, a Global Strategy for elimination of cervical cancer as a public health problem was drafted [33]. This calls for a comprehensive approach that includes prevention, screening and treatment, with a proposed global target that 90% of girls aged ≤15 years have been vaccinated for HPV by 2030. In a meeting of the WHO Strategic Advisory Group of Experts (SAGE) in October 2018, HPV vaccination was declared to be the most critical intervention for eliminating cervical cancer [34]. More recently, the World Medical Association announced its commitment to cervical cancer elimination, emphasising the need to improve HPV vaccination coverage [35]. However, the commitment to eliminate cervical cancer will be difficult to achieve without novel vaccination strategies to reduce HPV infection. A single dose schedule could help achieve this goal by reducing the cost and complexity of delivery.

In 2019, estimated global coverage among girls in the target range for vaccination (9–14 years) was 40%, and only 8–9% of 10-20-year-old girls have been vaccinated [36]. An estimated 30% of girls aged 9–14 years globally live in countries that have introduced the HPV vaccine, which means that many girls in the target age range for the vaccine are likely to remain unvaccinated [37]. The Tanzanian national HPV vaccination programme was rolled out in 2018, and is delivering 2 doses of the 4-valent vaccine (Gardasil®) to girls aged 14 years. However, coverage in 2019 was only 49% [unpublished data from the Tanzanian Ministry of Health provided to MITU/NIMR]. Furthermore, HPV vaccine supply has been constrained since 2018, which has affected HPV vaccination programmes worldwide, and supply is predicted to remain constrained for the next 3–5 years [37]. In their 2018 meeting, SAGE called for a comprehensive evaluation of options for the best use and allocation of the limited vaccine supply [35]. Given the large number of countries that have yet to adopt an HPV vaccination program, the lower cost and greater flexibility of a 1 dose HPV vaccination schedule has the potential to increase HPV vaccine introductions globally. The 1 dose schedule would also facilitate the introduction of the HPV-FASTER scheme, which proposes to combine HPV vaccination in women aged up to 30 years with at least one HPV-screening test, as a means to accelerate cervical cancer elimination [7].

Strengths of our trial are the comparison of two vaccine types, and 3 dosing schedules, allowing us to compare between/within vaccine types and dose schedules. Our outcomes focus on a full range of immune responses, including anti-VLP antibody levels, neutralising antibodies, antibody avidity, and memory B cell responses and the impact of malaria on these responses. There are no data on HPV vaccine antibody avidity or B cell memory from SSA, and no data on these functional aspects of the immune response for the 9-valent vaccine, so this will be the first trial to examine and compare these. We are also including a component to evaluate cost effectiveness and acceptability of the 1 dose schedule.

A limitation of our trial is that we are not collecting efficacy data because of the long duration of follow-up and large sample size that would be required, and because this is being done in the trial in Costa Rica with which we are harmonised (the ESCUDDO trial; NCT03180034). We are immunobridging to that trial and other earlier large efficacy studies in a variety of populations and settings, which will allow us to infer reproducibility of efficacy across different regions.

In summary, our trial will contribute robust evidence of the effect of the 1 dose schedule on a range of immune responses among young girls in SSA, and whether these may provide sufficient protection against HPV infection. The combined evidence from this and other ongoing 1 dose trials will provide critical information for policy-makers on the efficacy of this HPV vaccination strategy, which could alleviate vaccine supply constraints and expand access to the vaccine in the countries that need it most.

## Funding

The DoRIS trial is funded by the UK Department for International Development (DFID)/MRC/Wellcome Trust Joint Global Health Trials Scheme (MR/N006135/1) and the Bill and Melinda Gates Foundation (OPP1167526). KB and RH receive funding from the UK Medical Research Council (MRC) and the UK DFID under the MRC/DFID Concordat agreement, which is also part of the EDCTP2 programme supported by the European Union (MR/R010161/1). This project has been funded in part with Federal funds from the National Cancer Institute, National Institutes of Health, under Contract No. HHSN261200800001E. The content of this publication does not necessarily reflect the views or policies of the Department of Health and Human Services, nor does mention of trade names, commercial products, or organizations imply endorsement by the U.S. Government.
